# Serum markers of matrix turnover as predictors for the evolution of colorectal cancer metastasis under chemotherapy

**DOI:** 10.1038/sj.bjc.6600832

**Published:** 2003-04-15

**Authors:** B Hanke, A Wein, P Martus, C Riedel, M Voelker, E G Hahn, D Schuppan

**Affiliations:** 1 Department of Medicine I, University of Erlangen-Nuernberg, Germany; 2 Department of Gerontology, Humboldt University, Berlin; 3 Institute of Medical Informatics, Biometry and Epidemiology, Free University, Berlin; 4 Bayer AG, Leverkusen; Germany

**Keywords:** chemotherapy, CEA, CA-19-9, extracellular matrix, metastatic colorectal cancer, MMP-2, TIMP-1

## Abstract

Connective tissue turnover plays a prominent role in tumour growth and metastasis. We followed serum levels of seven connective tissue parameters in 37 patients with colorectal cancer metastatic to the liver prior to and during chemotherapy. Serum samples with episodes of tumour control (*n*=112) showed an increase of matrix metalloproteinase-2 (MMP-2) (*P*⩽0.01) and a decrease of tissue inhibitor of MMPs (TIMP-1) levels (*P*⩽0.01), while serum samples with episodes of tumour progression displayed the reverse pattern (*P*⩽0.01 and *P*⩽0.05, resp.). The ratio of circulating MMP-2/TIMP-1 was also significantly higher in episodes of tumour control *vs* tumour progression and prior to treatment (*P*⩽0.0001). We conclude that serum MMP-2 appears to reflect tumour resorption, while serum TIMP-1 may mirror tumour expansion.

Matrix metalloproteinases (MMPs) are proteolytic enzymes that play a central role in benign and malignant matrix remodelling (Kähäri and Saarialho-Kere, 1999; Nagase and Woessner, 1999). MMP-2 and MMP-9 (gelatinases A and B, resp.) degrade basement membrane collagen as well as denatured collagens (gelatin). MMP activity is tightly regulated (1) on the level of transcription, (2) on the level of proteolytic activation of the pro-MMPs, (3) by the tissue inhibitors of metalloproteinases (TIMP-1 and TIMP-2) and (4) by their strict compartmentalisation in cell membrane domains. Enhanced local expression and activity of MMPs and low levels of inhibitory TIMPs correlate with tumour growth and metastasis (Zeng *et al*, 1995,^1996^; Murray *et al*, 1996). However, the findings on circulating MMP- and TIMP levels in patients with metastatic colorectal tumours are unclear (Holten-Andersen *et al*, 2000; Pellegrini *et al*, 2000; Ylisirnio *et al*, 2000).

CEA and CA 19-9 have been previously shown to predict tumour growth in patients with liver metastasis treated by chemotherapy (Hanke *et al*, 2001). However, there exist no follow-up data with serum connective tissue markers to predict treatment response or nonresponse in these patients. Colorectal cancer metastatic to the liver is particularly suited to validate novel markers, since the condition is characterised by a large and defined tumour burden.

## MATERIALS AND METHODS

We studied 37 consecutive patients (29 men, eight women, age 42–76 years, mean 62 years) with colorectal carcinoma metastatic to the liver. Primary carcinoma was confirmed histologically. Histological confirmation was also obtained for synchronous liver metastasis. In case of metachronous liver metastasis, histological confirmation was only pursued when imaging techniques (spiral computerised tomography (CT) of the abdomen or MRT of the liver) did not show clear results. Patients received first-line chemotherapy, consisting of a weekly 1–2 h infusion of folinic acid (500 mg m^−2^) followed by a 24-h infusion of 5-fluorouracil (2600 mg m^−2^). One cycle comprised six weekly infusions followed by 2 weeks of rest. A total of 16 patients received additional biweekly oxaliplatin (85 mg m^−2^) and three patients additional weekly irinotecan (80 mg m^−2^) (Wein *et al*, 2001,^2003^). Treatment response was monitored every 8 weeks by spiral CT and antitumour activity was evaluated in accordance with WHO criteria. Median treatment duration was 7 months. Patients were stratified as ‘prior to treatment’, ‘tumour control’ (complete/partial remission or stable disease) and ‘tumour progression’. In 23 of 37 patients, first serum was obtained immediately prior to treatment. In all 37 patients, we recorded 112 episodes of tumour control, and in 16 of 37 patients we recorded episodes of tumour progression. The patients had been treated within the framework of two clinical phase II studies after obtaining approval by the local ethics committee (Wein *et al*, 2001,^2003^).

Sera were obtained at 8 weekly intervals, along with CT, stored frozen at −80°C and analysed for six circulating connective tissue markers in a single run. Serum collagen IV and VI, tenascin-C, MMP-2, the MMP-9/TIMP-1 complex and free TIMP-1 were measured on the Bayer Immuno 1™ Analyzer using fluorescein- and alkaline-phosphatase-labelled monoclonal antibodies to the target antigen (Schuppan *et al*, 1995; Ropers *et al*, 2000). Immune complexes were separated with magnetic particles coated with a monoclonal antifluorescein antibody and quantified after substrate addition. Analysis was restricted to MMP-2 and TIMP-1 (representing both the free and the bound protein), since only these markers correlated well with tumour evolution. Normal values for 100 healthy adults were 641 ng ml^−1^ (s.d. 5.5%) for MMP-2 and 620 ng ml^−1^ (s.d. 4.9%) for TIMP-1.

For descriptive analysis all sera referring to ‘prior to treatment’, ‘tumour control’ and ‘tumour progression’ were grouped together. For confirmatory analysis, values were averaged for each patient to yield only one value per category. With only four of the patients who contributed values for initial tumour status showing progression, confirmatory analysis was restricted to the comparison of ‘prior to treatment *vs* tumour control’ and ‘progression *vs* tumour control’. Comparisons were performed using the Wilcoxon test for paired samples. *P*-values were multiplied by two according to Bonferroni's correction for multiple testing. The level of significance was 0.05 (two-sided). All analyses were performed using SPSSWIN 9.0.

## RESULTS

A total of 37 patients received systemic chemotherapy during an average treatment duration of 7 months. Prior to treatment, serum TIMP-1 was elevated (>2 s.d. above the normal mean) in 57% (13 out of 23), decreasing to 36% (40 out of 112) in episodes with tumour control and increasing to 81% (13 out of 16) in episodes with progression (*P*<0.01 and *P*<0.05, resp.). Serum MMP-2 was elevated in 9% (two out of 23) prior to treatment, increasing to 35% (40 out of 112) in tumour control and decreasing to 12% (two out of 16) with progression (both differences *P*<0.01, see Table 1

**Table 1:** Numerical values of serum MMP-2/TIMP-1 in patients with metastatic colorectal cancer prior to and during chemotherapy

	**MMP-2 (ng ml^−1^)**				**TIMP-1 (ng ml^−1^)**				**MMP-2/TIMP-1**			
	**Mean**	**Median**	**s.d.**	**Range**	**Mean**	**Median**	**s.d.**	**Range**	**Mean**	**Median**	**s.d.**	**Range**
Prechemotherapy	670**	599	225	442–1439	956**	934	230	605–1318	0.73***	0.73	0.25	0.35–1.30
Tumour control	861	827	199	492–1396	808	752	228	454–1556	1.12	1.09	0.32	0.46–1.90
Tumour progress	744**	779	178	534–1130	1155*	1062	395	665–1949	0.71***	0.62	0.31	0.29–1.42

**Figure 1:**
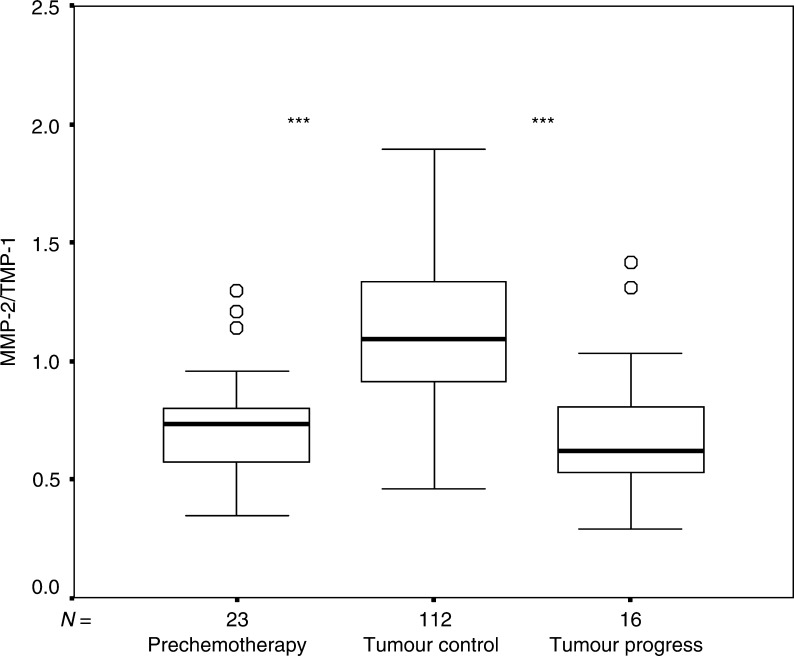
Ratio of circulating serum MMP-2/TIMP-1 in patients with metastatic colorectal cancer prior to and during chemotherapy. Circles correspond to extreme values in the statistical analysis. Total numbers of episodes=*N* (box plot analysis). Levels of significance compared with tumour control: ^*^P⩽0.05, ^**^P⩽0.01, ^***^P⩽0.001.

Levels of significance compared with tumour control:

* *P*⩽0.05,

** *P*⩽0.01,

*** *P*⩽0.001. Normal values: MMP-2 620 ng ml^−1^ (s.d. 4.9%), TIMP-1 641 ng ml^−1^ (s.d. 5.5%).

). The ratio MMP-2/TIMP-1 differed significantly between episodes and ranged from 0.73 ‘prior to treatment’, increasing to 1.12 under tumour control and decreasing to 0.71 upon tumour progression (*P*⩽0.001) (Figure 1

).

## DISCUSSION

This is the first report of the course of serum connective tissue markers in patients undergoing chemotherapy for colorectal carcinoma metastatic to the liver. Among the six tested markers, only MMP-2 and TIMP-1 correlated with the response to chemotherapy. Changes in elimination do not explain the differences in MMP and TIMP-1 serum levels, since during chemotherapy both serum creatinine and parameters of cholestasis remained unaltered.

The increase of MMP-2 and the decrease of TIMP-1 under tumour control is unexpected, since a common assumption is that enhanced activity of MMP-2, the classical basement membrane collagenase, leads to tumour growth and metastasis, while TIMP-1 as a crucial inhibitor of many MMPs should rather curtail tumour expansion (Nagase and Woessner, 1999). However, it remains to be proven if and how far tumour growth, which is accompanied by a strictly local control of proteolysis, leads to an excessive release of the involved enzymes or inhibitors (MMPs and TIMPs). Alternatively, an enhanced release of MMP-2, an enzyme which is sequestered on the cell surface and on collagens in the matrix, can be expected when the tumour volume and the associated desmoplastic stroma are reduced under effective chemotherapy. In addition, with enhanced resorption of tumour stroma, free TIMP-1 may be consumed by the various MMPs implicated in this process. The latter interpretation is in line with two cross-sectional studies in patients with advanced pulmonary and colorectal cancer, which showed elevated serum levels in tumours that progressed rapidly (Holten-Andersen *et al*, 2000; Pellegrini *et al*, 2000).

Therefore, therapies of advanced tumours that are based on unselective MMP inhibitors as a supplement to chemotherapy may be of little use, if not counterproductive. This assumption is supported by the disappointing outcome of several recent clinical studies using such agents (Bramhall *et al*, 2001; Coussens *et al*, 2002; Shepherd *et al*, 2002).
